# Evaluation of the phytochemical content, antimicrobial and antioxidant activity of *Cocos nucifera* liquid smoke, *Garcinia mangostana* pericarp, *Syzygium aromaticum* leaf, and *Phyllanthus niruri* L. extracts

**DOI:** 10.14202/vetworld.2021.3048-3055

**Published:** 2021-11-29

**Authors:** Tiurma Pasaribu, Arnold P. Sinurat, Elizabeth Wina, Triwardhani Cahyaningsih

**Affiliations:** Division of Nutrition and Agrostology, Indonesian Research Institute For Animal Production, Ciawi Bogor 16720, Indonesia

**Keywords:** antibacterial, antifungal, *Cocos nucifera*, extract, *Garcinia mangostana*, *Phyllanthus niruri* L, *Syzygium aromaticum*

## Abstract

**Background and Aim:**

Many plants contain bioactive substances with antibacterial and antifungal properties. The aim of this study was to evaluate the antibacterial and antifungal activity of *Cocos nucifera* shell liquid smoke (CSL), clove leaf extract (CLE), and mangosteen pericarp extract (MPE) alone and in combination against *Escherichia coli* and *Candida utilis*. The antioxidant activity, phenol, saponin, and tannin of CSL, CLE, MPE, and *Phyllanthus niruri* L. extract were also measured.

**Materials and Methods:**

The agar well-diffusion method was used to determine the antimicrobial and antifungal activities of CSL, methanolic MPE, and CLE and their combination CSL+MPE+CLE (COMBI) on bacteria *E. coli* and fungus (*C. utilis*). Antioxidant activity was measured by the diphenylpicrylhydrazyl method. Total phenol and total tannin were measured by the Folin–Ciocalteu method and total saponin was measured by the vanillin-sulphate method.

**Results:**

The results indicated that phenolic and tannin levels were greater in MPE than in CLE, whereas the saponin content was higher in CLE compared with MPE. Undiluted (100%) MPE exhibited lower antibacterial activity (p<0.05) than chloramphenicol against *E. coli*, however, undiluted CLE and COMBI showed similar activity compared with chloramphenicol against *E. coli*. COMBI caused significantly (p<0.05) higher inhibition compared with virginiamycin against *E. coli*. CSL, MPE, and COMBI exhibited significantly lower antifungal activity (p<0.05) than that of ketoconazole against *C. utilis*. In contrast, CLE showed improved antifungal activity (p<0.05) compared with ketoconazole.

**Conclusion:**

*Cocos nucifera* liquid smoke, *Garcinia mangostana* pericarp extract, and *Syzygium aromaticum* leaf extract, either alone or in combination, have the potential to be used as antibacterial and antifungal agents.

## Introduction

Agricultural feed additives are materials that do not contain nutrients, but aim to increase productivity, quality of livestock products (meat, eggs, milk, and fur), feed efficiency, and increase the immunity of livestock against disease. In some countries, feed additives that are widely used in the livestock industry belong to a class of antibiotics known as antibiotic growth promoters (AGPs). AGPs are added to minimize the population of pathogenic microbes in the digestive tract. In general, a provision of AGPs is to increase the growth of chickens by approximately 3.9% and the feed efficiency by 2.9% [1]. Since January 2018, however, the use of AGPs has been banned in Indonesia to avoid detrimental health effects to consumers, such as allergies and increasing number of drug-resistant microorganisms. European countries have banned the use of AGPs since 2006 [2]. Likewise, other countries, such as South Korea and the United States, have begun to limit AGP use. In 2019, there were more than 2.8 million people in the US suffering from infections, and there were 35,000 people who died from bacterial that was resistant to antibiotics [3]. There is a concern that this condition will increase if the use of AGPs is not controlled. In Indonesia, Noor and Poeloengan [4] reported that the use of antibiotics in animals was increased the resistance of Campylobacter and Salmonella bacteria to fluoroquinolone antibiotics and the third-generation antibiotics of cephalosporin. Antibiotics are generally given at a low dose (subtherapeutic level) at approximately 10-50 ppm [1]. Before AGPs were banned in 2018, the feed production in Indonesia was 18.2 million tons and it was calculated that the use of AGP would be around 182-910 tons/year. This amount will increase with the enhancement of feed production every year. However, this will increase the danger associated with resistance to antibiotics. However, the use of AGPs has been banned in Indonesia since 2018. Thus, an AGP alternative is needed, which is safer for livestock and the consumers of livestock products. In Europe, plant extracts have been used as a substitute for AGP [5]. Indonesia, as a tropical country, has a variety of plants containing bioactive compounds which may potentially serve as antibacterial and antifungal agents, such as *Psidium guajava* roots and leaves [6], cashew shell extract [7], mangosteen extract [8], *Plumeria rubra* flower and leaf extract [9], *Phyllanthus niruri* L. extract [10], and clove (*Syzygium aromaticum*) leaf extract [11].

We previously evaluated bioactive compounds from 12 plants and found three plant extracts that exhibit antibacterial, antifungal, and antioxidant properties (i.e., cashew nut shell liquid smoke, clove leaf extract [CLE], and mangosteen pericarp extract [MPE]) [12]. The combination of *P. niruri* L. and CLEs had the same ability to inhibit the growth of *E. coli* as the AGP, Zn bacitracin [13]. Although *P. niruri* extract exhibited the highest antioxidant activity, it is difficult to obtain enough quantity as it is a wild, non-cultivated plant. The MPE also contained high antioxidant activity [12] and is easier to obtain in large quantities. Other reports have shown that *Cocos nucifera* shell liquid smoke (CSL) also possesses antibacterial activities [14]. Since the coconut shell is abundant in Indonesia, the production of its liquid smoke is easier and cheaper than *Anacardium occidentale* shell liquid smoke. Therefore, a series of experiments was designed to produce a feed additive with antibacterial, antifungal, and antioxidant properties as an alternative to AGP consisting of the combination of CSL, MPE, and CLE (COMBI).

The aim of this study was to evaluate the phytochemical content, antibacterial and antifungal activity of CSL, mangosteen (*Garcinia mangostana*) pericarp extract, clove (*S. aromaticum*) leaf, *P. niruri* L. extract (PNE), and their combinations against *Escherichia coli* and *Candida utilis*.

## Materials and Methods

### Ethical approval

Ethical approval was not required for this study. All the experiments were performed *in vitro*.

### Study period and location

The study was conducted from October 2020 to March 2021 in Microbiology Laboratory of Indonesian Research Institute for Animal Production, Bogor, West Java, Indonesia.

### Preparation of extract and liquid smoke

Mangosteen *(G. mangostan*a) pericarp was collected from Purworejo-Central Java and clove leaves (*S. aromaticum*) and *P. niruri* L. were collected from Bogor, West Java. Both plants are very common in Indonesia and public of Indonesia can identify it easily on the basis of gross characteristics. Both plants were identified by the authors. The rind of the mangosteen was washed using water, drained, then cut into small pieces of approximately 0.5-1 cm^2^. Mangosteen pericarp, clove leaves, and *P. niruri* L. were dried in an oven at 60°C for 4-5 days. The dried mangosteen pericarp, clove leaves, and *P. niruri* L. were ground using a hammer mill, then screened using laboratory sieve No. 50 to obtain powder with a particle size of 300 microns.

One hundred and sixty grams of mangosteen pericarp, clove leaves, and/or *P. niruri* L. powder were mixed with 1440 mL of absolute methanol, placed in a shaker for 4 h, left to stand overnight at 26^o^C, and centrifuged at 10,000 RPM for 10 min at 4°C. The solution was filtered through filter paper and evaporated using a rotary evaporator at 40°C. This process produced the MPE, CLE, and PNE. *Cocos nucifer*a shell liquid smoke (CSL) was produced according to the procedure described by Pasaribu *et al*. [14].

A mixture of the three compounds, that is, CSL: MPE:CLE was made at a 1:1:1 ratio and this 100% stock mixture was designated an undiluted combination stock (COMBI).

### Antioxidant assay

The antioxidant activity of two ingredients, MPE and PNE, was measured. The antioxidant capacity was determined based on the percentage of diphenylpicrylhydrazyl (DPPH) radical inhibition and Vitamin C was used as the standard. DPPH was measured according to the method described by Rusmana *et al*. [15] with minor modification. Briefly, 1 mL of extract solution was diluted with sterile distilled water at various dilutions (8000; 12,000; 16,000; and 20,000 times) and mixed with 2 mL DPPH methanolic solution. The mixture was vortexed and left to stand in the dark for 30 min and the absorbance was measured at 517 nm. The radical scavenging activity was calculated by the following formula:

    Scavenging % = (Ac−As)/Ac×100

Where, Ac is the negative control absorbance (without sample) and As is the sample absorbance.

The results were presented as IC_50_ values (sample concentration required to inhibit 50% radicals). The lower the IC_50_ value, the higher the antioxidant activity of the sample [16].

### Phytochemical determination

Phytochemical (total phenol and saponin) content was measured for CSL, MPE, CLE, and PNE.

### Determination of total phenol

Total phenolic content was determined in CSL, MPE, CLE, and PNE using the Folin–Ciocalteu method [17]. Briefly, 0.2 g of each sample was diluted into 10 mL of acetone (70%) in a test tube, vortexed, and placed into an ultrasonic bath cleaner at −5°C for 20 min. The sample was diluted up to 50 times, 0.25 mL of Folin–Ciocalteu and 1.25 mL of Na_2_CO_3 (_20%) were added, mixed by vortexing, and allowed to stand for 40 min. The absorbance of the solution was read on a spectrophotometer at a wavelength of 725 nm.

### Saponin determination

Saponin content was measured in CSL, MPE, CLE, and PNE according to Hiai *et al*. [18]. Vanillin reagent (1.6 g) was added to 20 mL of absolute ethanol in a test tube and stirred. Plant extract (0.25 mL) was pipetted into a test tube, and 0.25 mL of vanillin reagent (fresh) and 2.5 mL 72% H_2_SO_4_ (in cold temperature) were added. The mixture was heated in a water bath for 10 min at 60°C and cooled. The absorbance of the mixture was measured at a wavelength of 544 nm. Diosgenin saponin was used as the reference standard.

Calculation : %Saponin


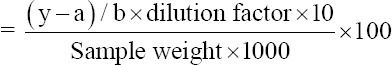


Where, y (absorbance); a and b (linear regression).

### Preparation of *E. coli* and *C. utilis*

*E. coli* was inoculated into nutrient agar (NA) media and incubated overnight for antibacterial tests. *C. utilis* was inoculated into potato dextrose agar (PDA) media and incubated for 5 days for the antifungal test. The turbidity of *E. coli* and *C. utilis* was measured using a spectrophotometer at a wavelength of 620 nm with a concentration compared to a standard (0.5 McFarland).

### Antibacterial assay

The antibacterial test was done by the agar well-diffusion method as described by Das *et al*. [19]. MPE, CLE, and COMBI were tested on NA media to measure the zone of inhibition against *E. coli*. *E. coli* was inoculated by spreading approximately 1 mL onto NA media with a cell count of 108 cells/mL, then leaving it for 4-5 min. The remaining liquid was pipetted and discarded. Four wells of 6 mm diameter were perforated into the agar medium with a sterile cork borer (6 mm) and filled with 60 μL of plant extract (MPE/CLE/COMBI) using a micropipette in each well under aseptic conditions. The MPE and CLE were diluted with sterile distilled water at different concentration, that is, 10%, 20%, 30%, 40%, 50%, 60%, 70%, 80%, 90%, and 100% (no dilution). Antibiotic (chloramphenicol, 18 ppm) was used as a positive control and sterile distilled water was used as a negative control. COMBI was also diluted to a range of concentrations (6.25%, 12.50%, 25%, 50%, 75%, and 100%). Two positive controls were established (i.e., virginiamycin 40 ppm [K^+1^] and chloramphenicol 30 ppm [K^+2^]).

### Antifungal assay

The antifungal test was carried out by an agar well-diffusion method as described by Ubulom *et al*. [20]. CSL, MPE, CLE, and COMBI were tested on PDA media to measure the zone of inhibition against *C. utilis*. *C. utilis* was inoculated by spreading approximately 1 mL onto PDA media with a cell count of 10^8^/mL, then leaving for 4-5 min. The remaining liquid was pipetted and discarded. Four wells of 6 mm diameter were perforated into the agar medium with a sterile cork borer (6 mm) and filled with 30 μL of plant extract (MPE/CLE/COMBI) using a micropipette for each well under aseptic conditions. MPE and CLE were diluted with sterile distilled water to establish a range of concentrations (10%, 20%, 30%, 40%, 50%, 60%, 70%, 80%, 90%, and 100%). Antifungal ketoconazole (30 mg/mL) was used as a positive control and sterile distilled water was used as a negative control. COMBI was also diluted to a range of concentrations (10%, 20%, 40%, 60%, 80%, and 100%).

Overall, the properties measured in this study included antioxidant activity; phytochemical analysis (total phenols, saponins, and tannins) of MPE, CLE, PNE, and COMBI; antibacterial activity of MPE, CLE, and COMBI against *E. coli*; and antifungal activity of CSL, MPE, CLE, and COMBI against *C. utilis*.

### Statistical analysis

All data were statistically analyzed using a one-way analysis of variance (ANOVA). Duncan tests were performed if the ANOVA showed significant differences (p<0.05). Assays were replicated 4 times and the values are expressed as the mean±standard error.

## Results and Discussion

### Antioxidant activity

Antioxidants are substances that can prevent or slow down damage to cells of organisms resulting from free radicals. A commonly used antioxidant is ascorbic acid (Vitamin C). IC_50_ values are commonly used as an indicator of antioxidant activity, which is the concentration of a substance that reduces DPPH free radicals by 50%. The lower the IC_50_ value, the higher the antioxidant activity [16].

The results showed that the IC_50_ value of *G. mangostana* pericarp extract was lower (0.05 μL/mL) compared with that of PNE (0.10 μL/mL) and the phenol content of *G. mangostana* pericarp extract was higher (12.09%) than that of PNE (3.78%) (Table-1). However, our previous results [12] indicated that the antioxidant capacity of PNE was slightly higher than that of MPE. The phenol content of MPE (12.09%) was higher than PNE (3.78%). This indicates that the phenol content exhibits a positive correlation with antioxidant activity. Plants that are rich in phenolic compounds are known to be good sources of natural antioxidants [21].

**Table-1:** Antioxidant activity (IC_50_) of MPE and PNE.

Source of antioxidant	IC_50_ (µL/mL)
MPE	0.05
PNE	0.10

MPE=Mangosteen pericarp extract, PNE=*Phyllanthus niruri* L. extract

### Phytochemical compounds

The highest total phenol and tannin content was found in MPE, whereas the lowest was in CSL. The highest saponin content was present in CLE (Table-2). Phytochemical compounds of *C. nucifera* shell liquid smoke consist of aldehyde, ketone, organic acid, alcohol, ester 2-propanone, acetic acid, methanol, 2-butanone, 2-propanone, 1-hydroxy-2 butanone, furancarboxaldehyde, phenol, benzene, and acetic acid as the most prominent [22]. This indicates that there were only a few phenolic compounds in CSL, which was in accordance with the results of the analysis (Table-2).

**Table-2:** Total phenol, saponin, and tannin content in CSL, MPE, CLE, and PNE.

Ingredients	Total phenol (%)	Saponin (%)	Tannin (%)
CSL	1.24	1.36	0.48
MPE	12.09	34.19	9.85
CLE	11.51	57.27	2.24
PNE	3.78	5.75	2.68

CSL=*Cocos nucifera* shell liquid smoke, MPE=Mangosteen pericarp extract, CLE=Clove leaves extract, PNE=*Phyllanthus niruri* extract

The pericarp of *G. mangostana* contains flavonoids, tannins, prenylated xanthones, benzophenones, bioflavonoids, triterpenes, and over 68 xanthone-type constituents [23-25]. The most prominent is α-mangostin [26].

Qualitatively, the CLE contains alkaloids, flavonoids, saponins, tannins, steroids, triterpenoids, and phenolics [27]. The main phytochemical components in clove extract were eugenol (63%–85%), eugenyl acetate (15%), caryophyllene (13.97%), and phenol, 2-methoxy-4-(2-propenyl), acetate (12.72%), and β-caryophyllene (5.12%) [28,29]. The aforementioned findings are in accordance with the results of the present study in which the phenol content in CLE was determined to be relatively low (i.e., 11.51%). Although the most abundant phytochemical in *P. niruri* was phyllanthin, the phenol content was low at 3.78%. To date, information regarding the tannin content of CSL, mangosteen pericarp, and clove leaves is limited.

Phenol, saponins, and tannins in COMBI at different dilution ratios were also measured to determine whether dilution had an effect on the phytochemical compounds. The phytochemical compounds of COMBI at a range of 10-100% showed that the higher the concentration, the higher the content of total phenols, saponins, and tannins (Table-3). Saponin content was higher compared with that of phenols and tannins in the COMBI. At all COMBI concentrations, the highest phytochemical content was saponins followed by phenols and tannins. This is in accordance with the levels in CSL, MPE, and CLE individually (Table-2) in which saponin levels were higher than phenols and tannins.

**Table-3:** Total phenol, saponin, and tannin content in COMBI with different dilutions.

Ratios	Total phenol	Tannin	Saponin
Dilution ratio	(g/100 mL)	(g/100 mL)	(g/100 mL)
1:9	0.11	0.07	1.17
2:8	0.27	0.17	1.52
3:7	0.28	0.19	2.25
4:6	0.69	0.41	2.36
5:5	1.15	0.80	3.10
6:4	1.54	0.99	8.41
7:3	1.45	0.96	10.07
8:2	1.98	1.08	11.54
9:1	1.96	1.09	14.19
Undiluted	1.52	1.02	14.60

COMBI=Combination of *Cocos nucifera* shell liquid smoke, mangosteen pericarp extract, clove leaves extract

### Antibacterial activity of MPE and CLE

**Figure-1:**
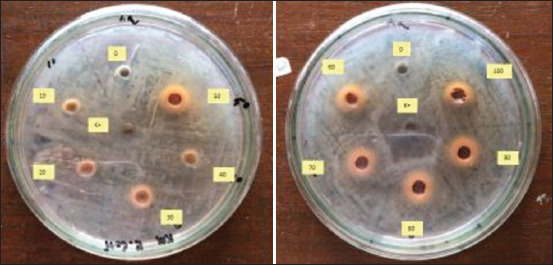
Inhibition zone of MPE at various concentrations against *Escherichia coli*.

**Figure-2:**
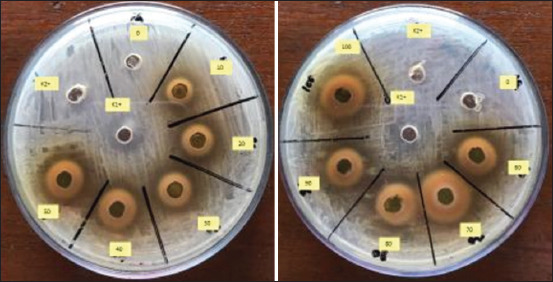
Clove leaf extract inhibition zone at various concentrations against *Escherichia coli*.

The antibacterial activity of MPE and CLE individually at different dilutions was determined against *E. coli* (Table-4 and illustrated in Figures-1 and 2). The inhibition zone resulting from MPE and CLE is shown by the clear areas. The results indicated that the dilution ratio significantly (p<0.01) affected the zone of inhibition against *E. coli*. There was no clear zone present when MPE was diluted from 1:9 to 4:6, but the clear zone appeared when MPE was present at higher concentrations (dilution ratio of 5:5 to undiluted). The zone of inhibition of undiluted MPE was significantly lower (p<0.05) than that of chloramphenicol (18 ppm), with a clear zone of 11.75 mm and 13.75 mm, respectively (Table-4). The zone of inhibition of MPE against *E. coli* was not significant (p>0.05) when MPE was diluted with distilled water at 6:4, 7:3, and 8:2 ratios, whereas a dilution ratio of 9:1 was not significantly (p>0.05) different compared with undiluted MPE (Table-4). This indicates that MPE at a concentration of 50-100% was able to inhibit the growth of *E. coli* with an inhibition zone of 9.75-11.75 mm. The results of this study are similar to that of Jacob *et al*. [30], who found the inhibition zone of *G. mangostana* pericarp extract to be 11 mm. However, Permata *et al*. [31] reported a higher inhibition zone of MPE at 15.8 mm.

**Table-4:** Inhibition zone (antibacterial activity) of MPE and CLE against *Escherichia* *coli* at different dilution ratios.

Dilution ratios (extract: sterile distilled water)	Clear zone (mm)	

	MPE	CLE
0 (sterile distilled water)	0.0+0.0^e^	0.0+0.0^g^
1:9	0.0+0.0^e^	8.00+0.33^d^
2:8	0.0+0.0^e^	9.00+0.33^cd^
3:7	0.0+0.0^e^	9.25+0.58^cd^
4:6	0.0+0.0^e^	10.25+0.88^bcd^
5:5	9.75+0.33^d^	10.25+0.88^bcd^
6:4	10.25+0.33^cd^	11.25+1.0^abc^
7:3	10.50+0.33^c^	11.75+0.88^ab^
8:2	10.75+0.33^c^	13.00+0.88^a^
9:1	11.50+0.00^b^	13.00+0.88^a^
Undiluted	11.75+0.33^b^	13.00+0.33^a^
Chloramphenicol 18 ppm (K^+^)	13.75+1.20^a^	13.50+0.58^a^
p-value	<0.001	<0.001

Different superscript^(a-e)^ within the same column indicates significantly different (p<0.05). Data were means of four replicates±standard error. MPE=Mangosteen pericarp extract, CLE=Clove leaf extract

The inhibition zone for CLE at a dilution ratio of 7:3 to undiluted was not significantly different (p>0.05) with those of chloramphenicol (18 ppm) against *E. coli*, with a clear zone of 11.8-13.0 versus 13.5 mm, respectively (Table-4). Unlike MPE, CLE exhibited growth inhibition of *E. coli* at a lower concentration from a dilution ratio of 1:9 to undiluted, with an inhibition zone of 8-13 mm. This result indicates that CLE had similar activity to 18 ppm chloramphenicol. The results of this study were lower for the ethanol fraction of CLE, with an inhibition zone of 16.07 mm against *E. coli*, but similar for the n-hexane fraction, with an inhibition zone of 13.61 mm, when compared with the results of Ramadhani *et al*. [27]. CLE at a concentration of 10-100% produced an inhibition zone against *E. coli* of 6.3-15.8 mm [11]. CLE at a concentration of 25% has also been reported to inhibit the growth of *Salmonella typhi* with an inhibition zone of approximately 16.90 mm [32].

At 100% concentration, the zone of inhibition of MPE against *E. coli* was approximately 11.75 mm, whereas CLE was around 13 mm (Table-4) and CSL was around 22.88 mm (in press). This indicates that CLE is stronger than MPE, but CSL was still more active than CLE against *E. coli*.

Bioactive substances (α-mangostin, eugenol, and acetic acid) kill bacteria by damaging the cell membrane as a result of the reaction between phenolic compounds and cell wall phospholipids. As a result, the permeability of the cell membrane is disrupted which inhibits the function of mRNA and bacteria development [33]. The acetic acid in CSL, which is protonated at low pH, permeates the lipid bilayer of the cell wall and releases protons into the intracellular environment [34]. Thus, more damage to the cell walls occurs compared with eugenol of CLE and α-mangostin of MPE. This indicates that acetic acid has a greater ability to inhibit the growth of pathogenic *E. coli* bacteria.

### Antibacterial activity of COMBI

**Figure-3:**
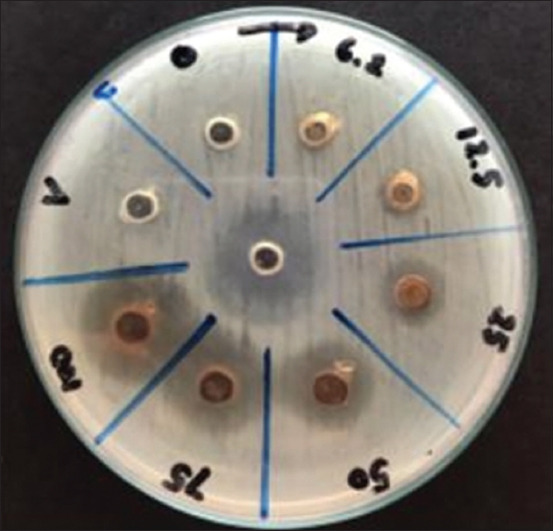
Antibacterial activity of combination of *Cocos nucifera* shell liquid smoke, mangosteen pericarp extract, and clove leaves extract at concentrations against *Escherichia coli*.

The antimicrobial activities of COMBI against *E. coli* at different concentrations are shown in Table-5 and Figure-3. The higher the COMBI concentration, the greater the zone of inhibition zone. Increased concentration of plant extracts causes greater cell membrane damage, so the inhibition zone is wider [35]. The wider zone of inhibition for 100% COMBI exhibited a greater destructive power because of the higher amount of bioactive substances penetrating into *E. coli* cells.

**Table-5:** Inhibition zone (antibacterial activity) of COMBI against *Escherichia coli*.

Concentration	Clear zone (mm)
0 (sterile distilled water)	0.0+0.0^g^
6.25%	6.71+0.18^f^
12.50%	9.21+0.26^e^
25%	12.86+0.28^d^
50%	16.14+0.50^c^
75%	17.97+0.05^b^
100% (no dilution)	19.50+0.19^a^
Virginiamycin 40 ppm	6.68+0.16^f^
Chloramphenicol 30 ppm	19.0+0.29^a^
p-value	<0.001

Different superscript^(a-g)^ within the same column indicates significantly different (p<0.05). Data were means of seven replicates±standard error. COMBI=Combination of *Cocos nucifera* shell liquid smoke, mangosteen pericarp extract, clove leaves extract

The efficacy of the antimicrobial activities of COMBI at a concentration of 6.25% was still able to inhibit the growth of *E. coli*, with an inhibition zone of 6.13 mm. COMBI at 100% concentration equaled the inhibition zone of 30 ppm chloramphenicol against *E. coli*, with values of 19.50 mm and 19.00 mm, respectively. COMBI was more effective (19.50 mm) than virginiamycin 40 ppm (6.25 mm) against *E. coli*. This indicates that a mixture of CSL, MPE, and CLE is able to inhibit the growth of pathogenic bacteria. The CSL inhibition zone at a 100% concentration was approximately 22.88 mm (in press). It was 11.75 mm and 13 mm for MPE and CLE, respectively. This shows that the CSL inhibition zone was still greater compared with COMBI if used alone. In contrast, MPE and CLE individually exhibited inhibition zones that were smaller than COMBI.

The reduced effectiveness of COMBI (the combination of the three plants) occurred because MPE at a concentration of 10-40% could not inhibit the growth of *E. coli*, whereas CSL and CLE at a concentration of 10% inhibited the growth of *E. coli*. The main chemical component of CSL is acetic acid, whereas it is α-mangostin in MPE and eugenol in CLE, which indicates that the combination could still inhibit *E. coli* growth. These findings also indicate that the bioactive substances in COMBI, which predominantly damage the cell membrane of *E. coli*, are acetic acid and eugenol.

### Antifungal activity of CSL, MPE, and CLE

The antifungal activity of CSL, MPE, and CLE on *C. utilis* is shown in Table-6. CSL at a concentration of 100% was significantly (p<0.05) less effective than ketoconazole at inhibiting the growth of *C. utilis* (7.50 mm vs. 16.75 mm) (Table-6). However, CSL was still able to inhibit the growth of *C. utilis* at a concentration of 80-100%, yielding a zone of inhibition of 6.50-7.50 mm. The predominant component, acetic acid, from CSL damages the cell walls of the *C. utilis* fungus so that its growth is disrupted. Using the microplate reader method, CSL inhibited the growth of *C. utilis* by up to 86.8% [14]. In contrast, there was no antifungal effect of CSL on *Candida* spp. [36]. This indicates that the antimicrobial activity of CSL was more effective on *E. coli* than *C. utilis*.

**Table-6:** Antifungal activity of CSL, MPE, and CLE and their combination in various concentrations against *Candida utilis*.

Concentration	Inhibition zone (mm)			

	CSL	MPE	CLE	COMBI
0 (sterile distilled water)	0.0+0.0^d^	0.0+0.0^g^	0.0+0.0^f^	0.0+0.0^d^
10%	0.0+0.0^d^	0.0+0.0^g^	6.38+0.13^e^	0.0+0.0^d^
20%	0.0+0.0^d^	8.0+0.41^f^	13.25+0.63^d^	0.0+0.0^d^
30%	0.0+0.0^d^	9.25+0.25^e^	13.75+0.63^d^	-
40%	0.0+0.0^d^	10.0+0.41d^e^	14.50+1.19^cd^	9.50+0.87^c^
50%	0.0+0.0^d^	10.25+0.48^cde^	15.50+0.29^c^	-
60%	0.0+0.0^d^	10.50+0.25^dc^	18.50+0.65^b^	10.75+0.48^bc^
70%	0.0+0.0^d^	10.75+0.29^dc^	19.75+0.25^ab^	-
80%	6.50+0.50^c^	10.75+0.25^dc^	20.75+0.25^a^	11.50+0.87^b^
90%	7.50+0.50^b^	11.25+0.25^c^	21.00+0.41^a^	-
100%	7.50+0.50^b^	12.50+0.65^b^	21.25+0.25^a^	11.75+0.48^b^
Ketoconazole (30 mg/mL)	16.75+0.63^a^	14.75+0.25^a^	16.0+0.25^c^	15.50+0.029^a^
p-value	<0.001	<0.001	<0.001	<0.001

Different superscript^(a-g)^ within the same column indicates significantly different (p<0.05). Data were means of four replicates±standard error. CLS=*Cocos nucifera* shell liquid smoke, MPE=Mangosteen pericarp extract, CLE=Clove leaves extract, COMBI=Combination of CLS, MPE, and CLE

Similarly, MPE at a concentration of 100% was significantly (p<0.05) less effective than ketoconazole at inhibiting the growth of *C. utilis* (12.50 vs. 14.75 mm) (Table-6). However, MPE at a concentration of 20-100% was able to inhibit the growth of *C. utilis*, with an inhibition zone of approximately 8.0-12.50 mm. The inhibition zone for MPE against *C. utilis* was not significantly (p>0.05) different at concentrations ranging from 40% to 90%. The effect of MPE on the inhibition zone of *C. utilis* was minimal at a concentration of 20%, which indicates that if the concentration was below 20%, MPE would be ineffective at inhibiting the growth of *C. utilis*. According to Geetha *et al*. [8], the inhibition zone of mangosteen fruit extract against *C. utilis* was 9 mm. Data on the effect of MPE on *C. utilis* have not yet become available.

The inhibition zone for CLE at 100% concentration was significantly (p<0.05) greater compared with that of ketoconazole (21.25 mm and 16.0 mm, respectively) (Table-6). CLE at a concentration of 10% was able to inhibit the growth of *C. utilis* with an inhibition zone of approximately 6.38. CLE inhibited the growth of *Candida albicans*, whereas clove oil exhibited robust antifungal activity [37,38]. Data on the effect of CLE on *C. utilis* are still limited.

The results showed that CLE exhibited the highest growth inhibitory effect against *C. utilis* when compared with MPE and CSL. The bioactive substance, eugenol, in CLE was more effective than α-mangostin from MPE and acetic acid from CSL to damage the cell walls and disrupt the growth of *C. utilis* fungi.

### Antifungal activity of the CSL, MPE, and CLE combination

The COMBI inhibition zone at 100% concentration was significantly lower (p<0.05) than that of ketoconazole (Table-6); however, COMBI was able to inhibit *C. utilis* (9.5 mm) at a minimum concentration of 40%. At a concentration of 80-100%, the COMBI inhibition zone (11.5-11.75 mm) was significantly (p<0.05) greater compared with a concentration of 40%. When each extract was tested against *C. utilis* individually, the minimum inhibition zone for CSL occurred at a concentration of 80%. The minimum concentration was 20% and 10% for MPE and CLE, respectively. This indicates that the effectiveness of CSL, MPE, and CLE was greater individually than in combination against *C. utilis*. Furthermore, α-mangostin from MPE and eugenol from CLE had an important role in inhibiting the growth of *C. utilis* in the COMBI treatment. This indicates that the activity of plant extracts is better when used individually compared to treatment of a mixture of several plant extracts for inhibiting *C. utilis* growth.

Apart from being antibacterial and antifungal, phenols exhibit other properties, including antioxidant activity. The mechanism through which bioactive substances kill bacteria is similar, namely, by damaging cytoplasmic cell walls and nucleotides and disruption the cell membrane to inhibit DNA and protein synthesis [39,40]. These results indicate that the combination of MPE, CLE, and CSL could be used as an antifungal treatment to inhibit the growth of *C. utilis*.

## Conclusion

The antioxidant activity of MPE was greater compared with that of PNE. Phenolic and tannin compounds were higher in MPE than in CLE, whereas the saponin compound was higher in CLE. Undiluted (100%) MPE exhibited a significantly lower antibacterial activity than chloramphenicol against *E. coli*; however, undiluted CLE and COMBI exhibited similar antibacterial activity compared with chloramphenicol. The COMBI at a low concentration (6.25%) showed similar antibacterial activity as 40 ppm virginiamycin; however, the antifungal activity of COMBI occurred at a concentration >40%. In summary, *C. nucifera* liquid smoke, *G. mangostana* pericarp extract, and *S. aromaticum* leaf extract, either alone or in combination, have the potential to be used as antibacterial and antifungal treatments.

## Authors’ Contributions

TP: Conceived and wrote the manuscript. TP, APS, and EW: Revised the manuscript and carried out the research work. TP and TC: Performed the statistical analysis. All authors read and approved the final manuscript.
